# Trends in treatment of anterior cruciate ligament injuries of the knee in the public and private healthcare systems of Brazil

**DOI:** 10.1590/1516-3180.2013.1314498

**Published:** 2013-08-01

**Authors:** Diego Costa Astur, Rodrigo Ferreira Batista, Arliani Gustavo, Moises Cohen

**Affiliations:** I MD. Orthopedist, Department of Orthopedics and Traumatology, Universidade Federal de São Paulo (Unifesp), São Paulo, Brazil.; II MD. Resident, Department of Orthopedics and Traumatology, Universidade Federal de São Paulo (Unifesp), São Paulo, Brazil.; III MD, PhD. Full Professor and Head, Department of Orthopedics and Traumatology, Universidade Federal de São Paulo (Unifesp), São Paulo, Brazil.

**Keywords:** Anterior cruciate ligament, Surgical procedures, operative, Anterior cruciate ligament reconstruction, Delivery of health care, Healthcare disparities, Ligamento cruzado anterior, Procedimentos cirúrgicos operatórios, Reconstrução do ligamento cruzado anterior, Assistência à saúde, Disparidades em assistência à saúde

## Abstract

**CONTEXT AND OBJECTIVE:**

Orthopedic surgery implies high costs for both public and private healthcare. The aim of this study was to better understand the differences between the public and private sectors regarding treatment of a damaged anterior cruciate ligament, which is a common knee injury.

**DESIGN AND SETTING:**

Descriptive cross-sectional study conducted during the Brazilian Orthopedics Congress in Brasília.

**METHODS:**

We applied questionnaires during the 2010 Brazilian Orthopedics Congress, with participation by 241 knee surgeons from 24 Brazilian states. This was followed by statistical analysis on the data that were obtained.

**RESULTS:**

The orthopedic surgeons who were evaluated used different approaches and treatment options in different Brazilian states, comparing between the public and private systems.

**CONCLUSION:**

Both in the public and in the private systems in Brazil, because of non-medical issues surrounding the treatment, the best medical decision is not always made. This may be harmful both to patients and to physicians.

## RESUMO

**CONTEXTO E OBJETIVO:**

Cirurgias ortopédicas implicam alto custo para a saúde pública e privada. O objetivo deste estudo foi de entender melhor as diferenças entre os sistemas público e privado em relação ao tratamento da lesão do ligamento cruzado anterior, que é uma lesão comum do joelho.

**TIPO DE ESTUDO E LOCAL:**

Estudo transversal descritivo, realizado durante Congresso Brasileiro de Ortopedia em Brasília.

**MÉTODOS:**

Aplicamos questionários durante o Congresso Brasileiro de Ortopedia de 2010, com a participação de 241 cirurgiões de joelho de 24 estados brasileiros. Posteriormente foi feita análise estatística nos dados obtidos.

**RESULTADOS:**

Diferentes condutas e opções de tratamento em diferentes estados brasileiros são realizadas pelos ortopedistas avaliados, quando comparadas em relação à atuação no sistema público e privado.

**CONCLUSÃO:**

No Brasil, tanto no sistema público como no privado nem sempre a melhor decisão é tomada por conta de efeitos outros que não a decisão médica, fato que tem sido maléfico para o paciente e para o médico.

## INTRODUCTION

The anterior cruciate ligament (ACL) is an important structure that stabilizes the knee by minimizing anterior translational and rotational instability.^1^^,^^2^^,^^3^^,^^4^ Injury to the ACL is extremely common among young patients and is usually secondary to high-energy and/or sports activity-related trauma.^5^^,^^6^

The ACL consists primarily of type 1 collagen fibers and extends from the inner face of the lateral femoral condyle to a fossa that is located anteriorly and laterally to the medial intercondylar tibial eminence.^1^^,^^2^^,^^3^^,^^4^ The ACL is composed of anteromedial and posterolateral bundles that are tense during flexion and extension, respectively.^1^

The ACL has a region of poor vascularization that is located 5-10 mm proximally to the tibial insertion with a transition area between the proximal sections. The blood supply to the ACL is via the medial genicular artery, and the distal section is supplied by the lateral and medial branches of the inferior genicular artery. This architecture confers low potential for healing in this region.^1^^,^^7^ Therefore, there is an international consensus within the field of orthopedics that surgical treatment is the best option for athletes and active people and should be performed within three weeks of the injury.^1^^,^^6^^,^^8^

In the United States, the incidence of ACL injury is rising: 80-100,000 reconstructions are performed annually, with an estimated associated cost of approximately 3 billion dollars.^7^^,^^9^^,^^10^^,^^11^^,^^12^ In Brazil, little epidemiological information is available regarding this injury or the reconstruction of the ligament. This issue has important social and economic implications, and there is no consensus in the field regarding the various issues that surround this therapy. As a consequence of this variability, decision-making regarding treatment differs widely among surgeons.^13^ However, how much of this divergence is due to individual experience and how much is due to the availability of potential resources are currently unknown.

## OBJECTIVE

The aim of this study was to investigate the broader epidemiological issues in Brazil that create barriers against surgical treatment of anterior cruciate ligament injuries. An additional aim was to highlight the discrepancies between what are considered to be ideal treatments and the treatments that are applied in practice within the public and private healthcare systems.

## METHODS

This descriptive study was conducted by administering a questionnaire to a sample of knee surgeons in Brazil. The questionnaire was prepared in such a way that it would be both highly comprehensible and simple. It consisted of 16 multiple-choice questions that covered a variety of topics, including the number of years of experience, the annual number of ACL reconstructions performed by the surgeon and several other factors relating both to treatment and to rehabilitation after ACL reconstruction (Appendix 1).

The questionnaire was administered to Brazilian knee surgeons during the three-day 2010 Brazilian Congress of Orthopedics and Traumatology. Only orthopedists who had previously performed ACL reconstruction surgeries completed the questionnaire. A total of 241 questionnaires were completed, of which 15 were excluded. Three of the questionnaires were excluded because the surgeon was from another country (Portugal, Bolivia and Peru), and 12 were excluded due to incomplete responses. A total of 226 questionnaires were fully completed and were used for subsequent analysis. Two researchers were available throughout administration of the questionnaires to answer questions from the participants in the Congress.

To test associations shown by the types of service variables (public or private) in relation to the other variables, we used the chi-square or Fisher exact test, as appropriate. The data were analyzed using the Statistical Package for the Social Sciences (SPSS) for Windows, version 16.0, at a significance level of 5%.

## RESULTS

During the 2010 Brazilian Congress of Orthopedics and Traumatology, 226 surgeons from 24 Brazilian states and the Federal District completed the questionnaire, which asked questions regarding their surgical practices within the public healthcare system and private healthcare system (or both) and their actions, in relation to 21 different topics.

The majority of the participant surgeons were from southeastern Brazil (54.4%), as show in Table 1. Two different groups comprised the sample: Group 1 was composed of 86 surgeons who operated predominantly within the public system; and Group 2 was composed of 50 surgeons who operated predominantly within the private system (Table 2). The remaining 90 surgeons in the sample performed surgeries with equal frequency within the public and private systems and were therefore excluded from further analyses.

**Table 1.** Region of origin of the study participants

	Southeast	South	Northeast	North	Center-West
State distribution (%)	54.5	12.4	15	6.6	11.5

**Table 2.** Activity in the public, private system or both among study participants

	Group 1 (Public)	Group 2 (Private)	Others
Surgeons	86	50	90
Years of experience	6.5	9.6	-

The first question related to the surgeon’s number of years of experience. In Group 1, the mean was 9.6 years of experience, and in Group 2, the mean was 6.5 years (Table 2). Statistically, there was no difference between the groups.

We analyzed and compared the data regarding the following: the surgeon’s area of expertise, length of experience, number of surgeries performed annually, type of graft used, mode of access, fixation used for femoral and tibial implants, technique used, use of pretensioning, length of surgery that was considered to be ideal, use of preoperative physical therapy, use of a rehabilitation protocol, use of an immobilizer after surgery, use of a drain, associated meniscal injury sutures and the time taken for patients to return to sports and/or activities. The results are shown in Tables 3, 4, 5 and 6 according to the area of activity: public or private systems (Groups 1 or 2).

**Table 3.** Time considered by the participant surgeon as the ideal between lesion and surgical treatment according to the area of activity: public (Group 1) or private (Group 2) sectors

Time for surgery (%)	Ideal	Group 1	Group 2
Up to 4 weeks	59.8	7.9	31.1
4-12 weeks	33.2	14.5	45.1
12-24 weeks	4	13.1	18.1
6 months to 1 year	3	64.6	5.7

**Table 4.** Surgical technique used by Brazilian orthopedic surgeons in anterior cruciate ligament lesion treatment, type of graft and type of implant used according to the area of activity: public sector (Group 1) or private sector (Group 2)

Surgical technique (%)	Group 1		Group 2	
Transtibial single branch	68		53	
Transportal single branch	22		21	
Double branch	10		12	
Type of graft used (%)	Group 1		Group 2	
Flexors	61.4		65.3	
Patella	35.7		31.3	
Allograft	1.4		0.8	
Type of implant used (%)	Group 1 tibia	Group 1 femur	Group 2 tibia	Group 2 femur
Metal screw	56	41	41.1	30.2
Absorbable screw	33.8	9.8	55	19.3

**Table 5.** Frequency of use of meniscal sutures for treating anterior cruciate ligament (ACL) injuries, among surgeons in the public sector (Group 1) and private sector (Group 2) in Brazil (P = 0.16)

ACL + meniscal suture	Group 1	Group 2
< 3/month	98 %	98.8 %
> 3/month	2 %	1.2 %

**Table 6.** Presciption of rehabilitation sessions and use of drain after anterior cruciate ligament surgical treatment in Brazil and time taken for recovery according to the area of activity: public sector (Group 1) or private sector (Group 2)

	Group 1	Group 2	
Rehabilitation (%)	86	90	P = 0.39
Drain	28	31.4	P = 0.7
Recovery time (%)			P = 0.55
4 months	6	1.1	
5 months	8	6.9	
6 months	52	55.8	
> 6 months	34	36	

## DISCUSSION

In Brazil, patient management in the public healthcare system differs notably from that in the private healthcare system. In some respects, this difference in management does not result in any harm; however, in other ways, this difference may result in irreparable harm both to patients and to surgeons. This means that non-medical interference in orthopedic surgeons’ decisions can lead to a need to use outdated surgical techniques, thus making the surgery and postoperative treatment harder.^14^

We observed that both less-experienced and more-experienced doctors work in the public healthcare system. The difficulties that are associated with health insurance companies can be considered to be one of the primary reasons for choosing public service, even among doctors with higher qualifications.

More than half of the orthopedic surgeons in Brazil believed that patients with a cruciate ligament injury should receive surgery within four weeks of the injury. However, only 5.4% and 26.5% of the surgeons in groups 1 and 2, respectively, performed surgery within this timeframe. The literature is not consistent with regard to the optimal time to perform this surgery. Historically, most studies showed that surgery should be performed within four to ten weeks of the injury.^7^^,^^8^ Today, the tendency is to focus on reducing edema and inflammation rather than focusing on the length of time since the injury. In practice, surgeons do not always perform the procedure within what they themselves consider to be the ideal timeframe. This is usually due to problems that are related to limited hospital resources, insufficient numbers of healthcare centers, budget reductions and the operational difficulties of the public system, all of which usually result in unmanageable queues and long waiting times for surgical procedures. As a consequence, more than 50% of surgeries took place more than six months after the injury.

In the private system, the explanation for the disparity between the ideal and actual times for performing surgery lay in the difficulty in obtaining authorization from the health insurance company to perform the surgery. As a result, 45.1% of surgeons operated 4-12 weeks after the injury.

### Surgical technique

The three most commonly used techniques for anterior cruciate ligament reconstruction were also performed by orthopedic surgeons in Brazil. Reconstruction using a transtibial single band was the most widely performed technique and was the preferred option for 68% and 62% of the surgeons in the public system and private systems, respectively. Reconstructions using the transportal single branch technique and the double branch technique were performed less often, although they have been reported to yield the best results.^7^^,^^15^^,^^16^ The limited use of these latter two techniques was likely due to a lack of availability of particular instruments and the higher cost of the larger number of implants that were required. Attempts to reduce public costs favor use of the transtibial technique in the Brazilian National Health System (Sistema Único de Saúde, SUS). In the private system, the difficulty in getting health insurance companies to accept the use of more costly techniques, despite better surgical outcomes, favors the contractor. Thus, patients and doctors do not obtain the best results from the anatomical reconstruction and reproduction technique.

The newly reconstructed ligament consists of either an autogenous tendon graft or an allograft. The graft type preference, as selected by the surgeon, was similar in both groups, and this was consistent with findings from other countries, including the United States.^6^ The preferred types of grafts were from the semitendinosus, gracilis and patellar muscle tendons. However, in other countries, using a cadaver graft is becoming more common due to decreased risks of disease transmission and rejection and, in particular, because it causes less damage to the patient during graft removal.^17^ However, because this is an expensive practice, there are few situations in which this treatment option is permitted in Brazil.

The type of implant that is used also generates many conflicts between doctor and auditors in both the public and the private systems.^17^ For example, the surgeon is not always permitted to choose which material to use. In the public healthcare system, metal interference screws were the most widely used type, whereas in the private system, use of absorbable interference screws prevailed. Interference screws are essential for graft fixation in the bone tunnel. However, metal interference screws have several disadvantages, including the possible need for their removal after treatment has been completed, and this involves another surgical procedure. In addition, these metal screws imply the risk of more pronounced graft wear, and this can lead to premature rupture of the graft.

The association between anterior cruciate ligament injuries and meniscal injuries is highly significant, reaching as high as 70% of the cases.^5^^,^^18^ The meniscus is a structure with little healing capacity in its central area, but good healing potential in its peripheral portion. When dealing with a central lesion, partial meniscectomy is indicated. However, when a patient has peripheral lesions, suturing is an option for preserving the meniscus. Suturing the injured meniscus can also reduce the complications that may follow meniscus resection (e.g. osteoarthritis), and it enables preservation of the structure’s function. In Brazil, a meniscal suture requires costly implants, which makes it difficult for surgeons to obtain the materials that are needed for surgery. This was true for both the private and the public groups. Even with the high incidence of this type of injury, in both groups, fewer than 2% of surgeons routinely performed this procedure. This equated to fewer than three times a year, which is one of the lowest rates among all the prominent countries in which orthopedics is practiced.

### Postoperative management

Studying the epidemiological profiles of orthopedic surgeons who perform reconstructive surgery on the anterior cruciate ligament involves analysis on information that is collected from the first contact with the patient and continues until the time the patient is considered cured and is discharged from care. Most orthopedic surgeons within the public and private systems prescribed a rehabilitation protocol and did not use a postsurgical drain. The time taken for patients to return to sports activity was similar for the two groups. Most of the orthopedic surgeons (86% and 91.8% in the public and private systems, respectively) gave their consent for patients to practice sports six months after the surgery, and this is consistent with the literature.

## CONCLUSIONS

Both in the public and in the private healthcare systems in Brazil, non-medical issues surrounding the treatment can cause difficulties in making the best medical decision, and this may be harmful both to patients and to physicians.
