# ERRATUM

**Published:** 2014-02-01

## ERRATUM

In the article “Trends in treatment of anterior cruciate ligament injuries of the knee in the public and private healthcare systems of Brazil”, published in the São Paulo Medical Journal, volume 131, issue number 4, 2013, the correct name of the third author is Gustavo Gonçalves Arliani and not Arliani Gustavo. The article should be cited thus: Astur DC, Batista RF, Arliani GG, Cohen M. Trends in treatment of anterior cruciate ligament injuries of the knee in the public and private healthcare systems of Brazil. Sao Paulo Med J. 2013 July 4;131(4):257-63. PubMed PMID: 24141297. 

